# Book Reviews

**Published:** 1824-05

**Authors:** 


### MEDICAL AND SCIENTIFIC BIBLIOGRAPHY.

Vexat censura . . . . . . Corvos.

Art. I.—*Practical Observations on the Removal of every Species and Variety of Cataract, by Hyalonyxis, or Vitreous Operation; illustrated by Cases. With Critical and general Remarks on the other Methods employed.* By John Bowen, m.d. Fellow of the Royal College of Physicians, Edinburgh; Member of the Royal College of Surgeons, London; Member of the Fiso-Critico of Siena and of the Arcadia of Rome; Corresponding Member of the Georgofili of Florence, &c. &c. &c.—8vo. pp. 120. Callow and Wilson, London, 1824.

During the greater part of the last year, the paucity of medical publications rendered it unnecessary for us to continue the bibliographical portion of our Journal, as we were enabled to give a tolerably extended account of the most important works as they appeared. But our literary wealth has flowed in so fast during the early months of this year, that we feel it incumbent upon us to give some account of the volumes which have been presented to us, whilst the gloss of novelty is upon them; a duty which, both in justice to the authors and the public, we do not think it right to delay, since a long time might elapse before we should find an opportunity of devoting an especial review to each separate article. Commencing, then, with the work first received, we have to present to our readers the details of a new operation for the removal of every species of cataract, proposed by Dr. Bowen, who has resided for some years on the continent, and who states that he has performed the operation, which he describes in the volume before us, upwards of 160 times during the last four years of his residence in Italy.

In a short preface, Dr. Bowen assures us that thirty, out of thirty-one, patients have been restored to sight by this operation, which he has termed *hyalonyxis*, from ὑαλος, *glass*, (the vitreous humor taking its name from that substance,) and νυσσω, *I pierce*. To obviate any objection that might be made to this name, in consequence of the vitreous humour being pierced also in the common operation of couching, his operation, he says, might be termed *posterior lenticular operation*, or *posterior capsular*; but, these being objectionable from their length, he has preferred the term hyalonyxis. It is an operation which may be performed without apprehension, and repeated without fear. Inflammation is of rare occurrence, provided proper precautions are used. So far the preface: with regard to the book itself, as a book, we must observe, that the type is very beautiful, and that, although printed in Paris, the errors of the press are rare, and of no material importance. One plate, descriptive of the operation, and the instrument used by the author, is annexed to the work.

The author commences with some congratulatory observations upon the progress that ophthalmic surgery has made of late years in this country; and he observes that, in a few years more, both physicians and surgeons will be considered as deficient in their profession, without a knowledge of that branch. The *constitutional treatment* forms the more noble and philosophical part, and our author thinks, if any distinction be made, that this falls rather to the share of the physician than surgeon. We are quite sure that this distinction cannot be made, and that no very effectual or nice line of demarcation can be established between the two branches of the profession. Is syphilis, for example, a medical or surgical complaint? The primary symptoms may surely be claimed by the surgeon; but then, we suppose, the physician must have the patient when pains in the bones and eruptions are only to be detected: and, if this be objected to, what is to prevent the surgeon from claiming small-pox as his own? In a general way, the two branches of the healing art are sufficiently separated, and custom has assigned to each party a tolerable list of infirmities to attend to.

Our author observes that, notwithstanding the operation for the removal of cataract has been performed for so many ages, it is remarkable that not *one* fixed or determined method has been adopted as a guide to the young surgeon. Extraction, depression, keratonyxis (or the operation through the cornea), and merely breaking down the lens, and suffering it to be absorbed in situ, have each their advocates; but this latter operation is only adapted to soft or fluid cataracts. These differences of opinion, and the infinite subdivisions of the cataract, according to its real or supposed nature, have consequently contributed to render this subject much more complex; and, therefore, the operation proposed by Dr. Bowen must indeed be a great improvement, if it be found to answer the description he has given of it, as it is equally applicable to every species of this disease. It appears that diseases of the eye are more common in Italy than in any other country, with the exception of Egypt, and that, in Sienna, our author found the proportion of eye-cases to other diseases to be as one to eight; and at Rome, one in fifteen.

Passing over the causes of cataract, on which we find little said, and that little not very satisfactory, we next proceed to notice Dr. Bowen's opinion relative to the ripeness of a cataract for the performance of an operation; a distinction, the propriety of which he altogether denies, and upon what appears to us to be very good grounds; and he has no favourable opinion of any of the remedies which have at various times been extolled for the removal of this complaint: neither does he consider it necessary to wait until the opacity is complete. Provided inflammation of the lens does not exist, there can be no objection, he thinks, to the operation: consequently, he advises it to be performed where the person is only half-blind, because, "the earlier the operation, the more perfect is vision; for the retina, long unaccustomed to the stimulus of light, must inevitably lose more or less of its sensibility," (page 13.) But our author expressly says, in the next page, that, although this plan be not objectionable under circumstances of necessity, he does not usually recommend the operation before the opacity of the lens be complete. Now, granting that the reason with respect to the sensibility of the retina carries any weight with it, we are rather surprised to find that he should thus abandon the strong position he had just taken up. He again repeats this opinion immediately afterwards, that a diseased lens cannot too soon be got rid of, (page 15,) when affecting one eye only; a point which is still contested, as he observes, by many of the most eminent of the profession of the present day: and he recommends this the rather because, from sympathy, one eye is likely to become diseased in consequence of the existence of a cataract in the other; which was illustrated in one of his patients, (the Padre Bora,) who had a cataract removed from his right eye, of six or seven years' standing, the opacity of the left having just commenced; but, upon the removal of the lens of the right side, this opacity totally disappeared without an operation.

Dr. Bowen justly lays great stress upon the treatment, both prior to and after the operation; and especially in the former case, even when the patient is in the best health, he recommends the depleting plan to be strictly adopted, and followed up by the use of purgatives. He has rarely advised blood-letting, but recommends a pediluvium at bed-time; but, if there be any amaurotic affection conjoined, under which circumstances inflammatory symptoms are always to be dreaded, the preparation of the patient for the operation must be strictly attended to.

After some preliminary remarks on the instruments usually employed in this operation, Dr. Bowen proceeds to describe that which he uses.

"The human eye may be considered about nine lines and a half in diameter, passing transversely across, three lines and a half distant from the transparent cornea.

"The needle I employ is one line less than the diameter of the globe; so that a young operator, penetrating the sclerotic three lines distant from the transparent cornea, which is the distance recommended for hyalonyxis, or posterior capsular operation, hereafter to be described, can be under no alarm; for, admitting he carried the instrument as far as the handle, the opposite side could not be injured: he is far removed from the iris and ciliary processes, the parts most sensible and liable to inflammation when wounded,—the too-frequent causes of failure. In fact, he can do no mischief with his instrument in this situation.

"The instrument is lancet or sharp pointed, more slender than Scarpa's, and, from being so much shorter, has sufficient firmness, inflicting of course less injury; has two cutting edges near its extremity, which assists the operator in cutting and detaching any adhesions; slightly convex, but enters the sclerotic with the same facility as a straight needle. The diameter is uniformly the same from the cutting edge to the handle; so that, when you have punctured the sclerotic, &c. you advance and retire as you wish, and without any loss of vitreous humour, which the late Mr. Hey, of Leeds, seemed to be apprehensive of: to guard against which, he recommended his wedge-shaped needle, which I found extremely inconvenient during the operation; for, if you wish to advance, you are obliged to press the instrument forwards, so that the eye revolves towards the nose. Whereas, the operator will find it much more to his advantage, in facilitating his operation, to advance and retire at pleasure, without changing the position of the eye. The shortness of the needle gives the operator a quick command of its point, and he can use it with greater confidence than a needle of double its length. The needle is shorter than any in use in the present day; but it must be recollected that, in the operation for the removal of the lens, there are not more than five lines of the instrument in the globe, so that there still remains three lines and a half of the needle unemployed." (p. 26—28.)

The operation by hyalonyxis is thus performed; three or four hours previous to which, the patient's eyebrows should be rubbed with some of the extract of belladonna, rendered soft by the addition of a little boiling water.

"The patient being seated, so that the superior part of the head does not reach higher than the superior part of the sternum of the operator, he takes his needle, previously besmeared with oil, as a pencil or writing-pen, placing the weight of his hand on the cheek of the patient: this produces two very considerable advantages; first, it steadies the hand; second, in the event of the patient's moving, the hand preserves its relative position with respect to the eye, so that any movement of the patient's head can be of no importance to the surgeon. The patient being directed to turn the eye towards the nose, the needle, with its convex surface* forwards, corresponding with the iris, is boldly introduced into the globe of the eye through the sclerotic, three lines or three and a half from the transparent cornea, and a line below the transverse diameter of the pupil, to avoid wounding the ciliary artery, which pursues its course to the iris between the sclerotic and choroid coats, along the middle of the external convexity of the eyeball. If introduced at or below this point, you avoid all effusion of blood. The instrument passes into the vitreous humour posterior to the lens and its capsules; the point of the needle is then brought forwards, from inclining the handle to the temple, and penetrates the posterior capsule.

"Should the lens be soft or fluid, the needle passes through its centre on its passage to the anterior chamber; if solid, the instrument should be directed to its superior part, and, by gently elevating the handle, the lens will be sufficiently depressed to allow the instrument to pass between it and the ciliary processes; it is then carried through the anterior capsule and pupil, (which latter has been previously dilated with the belladonna,) into the anterior chamber, without any risk or danger of wounding either ciliary ligament, iris, or ciliary processes. The object the operator has in view is the laceration and removal of a large portion of the anterior capsule,—that is to say, of greater extent and diameter than the pupil in its natural, most dilated state: this is effected by three or four circular movements of the point of the needle, which should invariably be done. If the capsule be transparent, the inexperienced operator will scarcely be sensible of effecting any good or change by this circular movement of the needle.

"The correspondence in transparency and colour of the capsule to the aqueous humour is so similar, and the former so delicate, that no resistance of the needle is sensible to the fingers, when in reality the point is breaking down and removing from the axis of vision the membrane, so frequently the cause of secondary cataract.

"When the capsule is opaque, the effects of the needle are of course evident. The transparent or opaque portions should be conducted backwards and downwards with the lens, and buried in the vitreous humour below the margin of the iris, and as much as possible removed from the front of the pupil. On retiring or withdrawing your instrument, a circular movement is similarly made with the point, for the purpose of removing every portion of the posterior capsule, which is also liable to opacity; so that a communication is completely established between the aqueous and vitreous humours. The anterior capsule, lens, and posterior capsule, are now removed; so that the utter and total impossibility of secondary membranous cataract forming, must be evident.

"Such is the operation for a solid cataract enveloped by its capsule; but the lens we usually meet with, at least seven in ten cases, is either fluid, semisolid, or curdy. When the instrument, therefore, penetrates the anterior capsule, and the operator commences his delicate circular movement, the aqueous humour becomes clouded or discoloured, which is the passage of parts of the diseased lens into the anterior chamber, where its absorption is more rapid than in the vitreous humour.

"The views of the operator are the same: instead of quickly finishing the operation, the solid particles of the lens should be removed by several movements of the needle as much as possible from the axis of vision, either by depressing them into the vitreous humour, or bringing them into the anterior chamber. The free movements of the needle, and the removal of the capsules into the vitreous or aqueous humour, as we have above described, ensure the success of the operation.

"The great desideratum in all fluid cataracts is the laceration and removal of the capsules from the axis of vision; for, in all cases, when a free communication is once established between the aqueous and vitreous humours, absorption goes on, and your cure is almost certain.

---

* To avoid still more the wounding of the ciliary nerve or artery, the needle may be introduced with its cutting edge corresponding with the iris; and, after having entered the eye, to be turned round with its convex surface forwards, and continued as we have before described.

"We have now described the removal of the lens in two different states; there only remain some observations on the third.

"I have occasionally met with the lens united firmly to both capsules, forming as it were one body. These cataracts, from their size and appearance, are easily distinguished from what Scarpa very properly terms primary membranous cataracts; which is the wasting of the crystalline within its capsule, which appears contracted and more opaque in its centre than common cataract. Tne most correct account, however, we have of this variety is from the late Mr. Saunders, of London, published by his friend and colleague, Dr. Farre, an eminent physician. Those cataracts are for the most part congenital, and, although Scarpa represents them as rare, are not uncommonly met with in children. When the lens in these cases is absorbed, the anterior lamella of the capsule unites itself to the posterior, until they form one membrane, which is white, opaque, and elastic: this membrane is absorbed after puncture with the needle and partial laceration. A most interesting account of congenital cataracts is given by Mr. Saunders, with their removal in children." (p. 42—48.)

We have only to remark, in closing this imperfect notice of Dr. Bowen's work, that he shows himself to be intimately acquainted with the writings of the best authors on ophthalmic surgery, both English and continental; and that he has made many sensible remarks on the diagnosis and prognosis of this malady, but which we do not deem it necessary to dwell upon, as our object is principally to give our readers a just notion of the particular operation recommended by the author as applicable to every form of the disease. With respect to the treatment after the operation, the total seclusion from light is said to be of the first importance. The strictest plan of diet is urged until the sixth day, a purgative being given on the morning succeeding the operation. After the sixth day, the living may be as usual; but the eye should never be examined until the twelfth day: the light is then to be gradually admitted into the patient's chamber to the twenty-fifth day, and after that time his ordinary occupations may be resumed. Should any inflammatory symptoms arise, the usual treatment in such cases must be resorted to.

The volume concludes with the detail of several very successful and interesting cases.

---

ART. II.—*Symptomatology; or, the Art of detecting Disease: a Lecture occasionally read to the Pupils of the Westminster Hospital, and published according to their request.* By ALEX. P. BUCHAN, M.D. F.L.S. late senior Physician to that Institution; and Member of the Royal College of Physicians, London. *To which are added, Tables of Symptoms.*—12mo. pp. 190. Callow and Wilson, London 1824.

WE have so frequently been obliged to toil through a heavy octavo, devoted to the consideration of one disease, nay, sometimes to one symptom of a disease, that it was quite refreshing to us to contemplate the little work now before us, comprising an account of the symptoms of all diseases; affording a specimen of the art of compression, which authors of the present day are not much in the habit of practising. The title-page informs us that this is the substance of a Lecture read to the pupils of the Westminster Hospital; and, as such, it must only be considered as a kind of text-book to the young practitioner,—a manual of hints, or subjects of reflection,—a set of sketches, in fact, which he must fill up and extend by careful observation at the bedside. It is not our intention to go through the examination of this work *seriatim.* We shall say, generally, that there are several remarks thrown out, which are well deserving of the pupil's attention; some reflections that appear novel; but there are also not a few points that call for criticism, and some that require explanation. We shall give a specimen or two of both sorts; and, as our praise and censure are given with equal sincerity, the author will, we trust, perceive that we have read his preface, and allow the force of his deprecatory appeal from the severity of criticism.

In the first place, we think that the discussion relative to the antiquity of the two branches of the healing art, and the story told in the note, had better been omitted. We are neither free-thinkers nor saints; but these are subjects which, professionally, we ought to avoid; especially when addressing students, who are, generally speaking, rather too prone to be *facetious* upon these topics, without prompting. Neither can we admit that the sister-arts of medicine and surgery have advanced with equal steps, inasmuch as the latter has by far outstripped the former in the race; though, from the nature and objects of her pursuit, this may be said without any imputation against the science of medicine. We have elsewhere given our opinion as to the impossibility of very strictly defining the limits of the two branches of the profession: in a general way the line is sufficiently marked, and any attempt to define it more exactly would perhaps only tend to confound that which has no real foundation in nature.

In pages 19 and 20 are some very judicious hints relative to the mode of examining a patient, and inquiring into disease.

The first symptom treated of is *pain,* the consideration of which extends through a space of upwards of ten pages, and affords scope for a great deal of reflection: the pupil should study it attentively, for most of the remarks are well-founded, though perhaps the observations are expressed a little more loosely than we could have wished. We extract the following paragraph as a useful caution to the young practitioner:

"The impropriety and indecorum of a physician, immediately on entering a sick chamber, seizing the arm of an invalid, and rudely applying his hand to the pulse, especially if a stranger to the patient, is abundantly obvious. The very presence of a medical man often agitates the nerves of a sick patient so much as to occasion material aberrations from the real state of the pulse. Conceiving it impossible to offer more correct instructions for the conduct of a physician on such an occasion, than those laid down by Celsus, I shall translate the passage from the work of that elegant writer.

"'It by no means,' says he, 'becomes a skilful physician, as soon as he enters the apartment of the sick, to seize the hand of the patient. He ought preferably to sit down, and with a calm and compassionate countenance to inquire concerning his usual state of health; and, if he perceives him to labour under any degree of timidity or depression of spirits, he should endeavour to sooth his mind by kind and encouraging conversation. After a proper interval, he ought, in a quiet and gentle manner, to apply his fingers in succession to the carpus. How frequently,' he adds, 'does the very sight of a physician perturb the pulse, the rhythm of which is liable to be deranged by a variety of slight circumstances.'" (p. 42, 43.)

In general, we may observe that the observations relative to the pulse are extremely good; and we may say the same of the remarks upon the appearances of the urine, a subject not so much attended to as it deserves, this excretion affording, in many diseases, very important information to the experienced practitioner. We insert the following hints, as worthy attention:

"Unnecessarily to multiply our questions, is always distressing to the patient. Irritability of temper is generally the concomitant of disease; and I have often observed, that sick persons will frame such replies as you seem to expect, rather than be teased with a protracted examination. This is probably one reason why many persons resort to those who pretend to discover the nature of diseases from the inspection of the urine alone, without asking any questions. Patients are, moreover, prone to consider too many questions as implying ignorance of the nature of their complaint. A lady once asked me, in a peevish tone, how those persons, who cured horses and dogs, found out what was the matter with them. We should, therefore, carefully endeavour to make ourselves acquainted with every obvious indication of disease, that may tend to diminish the necessity of troubling our patients with a multitude of interrogatories." (p. 79, 80.)

At page 90 we find it asserted, as an unfavourable omen, when the countenance of a sick person assumes a strong resemblance to that of either parent, not, perhaps, observable during health. We notice this because we believe it to be true only in the last stages of chronic disease, and simply the effect of emaciation, so that the contour of the countenance becomes changed, the plumpness and freshness of health and youth is removed, and the resemblance to the aged and wrinkled parent becomes visible. Thus, if our explanation be true, as an *omen* this change of feature is of no importance.

The next paragraph also requires some consideration. "Dropsy and diabetes, it is said, are sufficiently obvious to the senses of sight, touch, and taste." With regard to dropsy, we would observe, that, in the young female, it is necessary to recollect that pregnancy may be attempted to be concealed under the guise of dropsy, and that the operation of paracentesis has often been proposed, and sometimes performed, under these circumstances.

There is much good sense in our author's critique upon the fashionable doctrine of biliary disease and liver affections; and we agree with him fully in believing that a majority of the cutaneous eruptions met with arise from that source.

We have but one more criticism to offer, and that applies to the proposition of our author to make the bodies of those persons who die in hospitals, and who have no friends to defray the expence of their funerals, available for the purposes of dissection. We must enter our protest against this opinion, because, although the parties may be too poor to pay for the burial of their relatives, their feelings are equally deserving of our attention, and their prejudices must necessarily be greater than those of the more fortunately circumstanced; and we cannot but think that such a regulation would be a cruel addition to the already grievous burden which the sick poor have to contend against.

The remainder of this volume is composed of a Table of Symptoms, alphabetically arranged, which occupies nearly ninety pages; and there is a Plate, representing the different perceptions indicated to the sense of feeling by the pulse. This is copied from the "Semiotice Pathologica" of GRUNER, and it consists of eleven figures or diagrams; but we confess we do not see what possible advantage they can yield to the practitioner: we are quite sure, indeed, that we, at least, do not understand what they mean. We have only to observe, in conclusion, that Dr. Buchan's little work will, in our opinion, be useful to the medical student, by supplying him with hints, and pointing out the road to many important inquiries, which might otherwise have escaped his observation.

---

ART. III.—*An Essay on an improved Method of Cutting for Urinary Calculi; or, the Posterior Operation of Lithotomy.* By W. W. SLEIGH, Member of the Royal College of Surgeons, and Lecturer on Anatomy and Surgery in London, &c. &c.—8vo. pp. 104. John Anderson, London, 1824.

NOTHING can be more modest than the manner in which Mr. SLEIGH introduces this little work to the notice of the profession. The operation which he describes appears to be, as far as he is concerned, original, as he was not aware, at the time he first proposed it, that an operation in some respects similar had been performed in Europe. Our author was at that time residing in Canada, where he conducted an anatomical theatre. We shall presently see the essential difference between Mr. Sleigh's operation and that recommended and performed by Professor VACCA and M. SANSON; and our author observes, that, if the method proposed by those gentlemen is found successful, there can be little doubt of the superior advantages of his plan. It is not our intention to follow exactly the order and arrangement of Mr. Sleigh's work: it is not, perhaps, the best that might have been adopted, and it is also evident that the author is not much accustomed to literary composition; but these are minor considerations, and we should, perhaps, not have mentioned them, but it must be remembered that "we are nothing, if not critical." It will be urged by many, that we want no new operation for lithotomy; that the lateral operation is not open to the objections usually urged against it, if proper precautions be taken, and attention be paid both before and after the performance of it; and certainly it must be admitted, that the success that has attended some of the provincial surgeons, especially those of Norwich, is so great, as to leave little to be hoped for by any change of system in their hands. We say this without any suspicion of blame attaching to less fortunate operators, because we conceive that the state of preparation of the patient, the air which he breathes, and other circumstances, are to be considered as the causes of this astonishing difference in the result between town and country operations for lithotomy, and that the mere skill in performing them has little or nothing to do with it. However this may be, it is certain that the lateral operation is one of great danger; that the proportion of deaths (in public institutions, especially,) after the operation is very considerable; and that therefore, on this account merely, an improved method of extracting calculi from the bladder is a great desideratum in surgery.

We now return to Mr. Sleigh's work, the introduction to which consists of thirty-seven pages, and contains a pretty long account of the various methods of performing lithotomy, from the time of CELSUS inclusive, and which we may be excused for passing over, as most of our readers are, or ought to be, familiar with the various details. The first chapter of the work itself is devoted to a consideration of the necessity of a preparatory regimen, and which is doubly necessary in this instance, as far as emptying the intestinal canal is concerned; and afterwards endeavouring by opium to slacken the peristaltic motion of the bowels, and to induce constipation: this should only be done on the morning of the operation. An abstinence from solid food, particularly animal matter, is also strictly enjoined.

We now come to a description of the operation itself. The part of the bladder which Mr. Sleigh proposes to open "is its inferior posterior surface, which is destitute of a peritoneal tunic, and is only separated from the intestinum rectum by cellular substance: its shape is triangular, the base of which is placed backwards and upwards, and its apex forwards and downwards; its length is from one to two, or two and a half inches. It is bounded laterally by the vasa deferentia and vesiculæ seminales, superiorly by the cul-de-sac of the peritoneum, and inferiorly by the prostate gland, and union of the seminal tubes.

"The instruments I have invented for this operation are only two, (with a trifling alteration in the lithotomy forceps;) one for dilating the sphincter ani muscle, the other for making the incision into the bladder. The former I term the speculum ani, or dilating forceps; it is divided into its blades and handles, which go off from each other at a right angle. The blades are convex, and smooth externally, and are gradually reduced in breadth towards their extremities, so that, when closed, they occupy but a very small space, and thus may be introduced into the anus with the greatest facility, and without producing any uneasiness to the individual. A screw transfixes one of the handles (its right one), and rests upon the internal surface of the other; by the turning of which the blades are slowly, steadily, and gradually separated, and full command is obtained over the sphincter ani, which becomes dilated in proportion as we turn the screw, and consequently to any admissible extent we may desire.

"The staff may be of the ordinary description; its size and curve are to be regulated according to circumstances, as the age of our patient, &c. The scalpel is seven inches in length; its blade is one inch and a quarter only, which is concealed in an open sheath, that is attached to the root of the blade by a steel spring; by which means the edge of the knife is entirely and firmly covered, until pressure be made with it on some soft substance, as if in the act of cutting; when, the sheath retiring back in proportion to the force used, the blade becomes naked, and, the incision being effected, the sheath instantaneously recovers it, in consequence of its spring. A few different-sized lithotomy forceps must be at hand for the purpose of extracting the stone, should it not be thrust out on the incision being made.

"The state of the constitution having been regulated according to the directions mentioned in another part of this work, and an enema administered, the patient is put in the same situation as for the lateral operation. The speculum ani (having lain in a little warm water for some time previously, and then rubbed with a little sweet oil,) should be gradually introduced into the rectum; the screw should then be slowly and steadily turned, by means of which its blades are separated, and the power of the sphincter ani muscle overcome. The anus thus opened to a sufficient extent* (transversely,) the index-finger of the left hand should be placed on the posterior edge of the prostate gland, which is the anterior boundary of the part of the bladder to be divided. The scalpel is then to be introduced, and, by measuring the knife with the index-finger, the length of our incision can be regulated with accuracy, according to our wishes. The staff having been previously introduced into the bladder, will be felt pressing the coats of the viscus against the intestinum rectum. This will be an infallible criterion by which we can judge of the situation for our incision, and of the nature of the substance interposed between the staff and our finger. We can then divide the parts

* "We may safely dilate it from two to three inches in an adult."

either directly on the staff or by its side. Upon the incision being made, the urine will gush out, and in all probability the stone will be forced out in the same moment. Should the calculus not escape with the urine, the common forceps is to be introduced through the rectum into the bladder, and the stone extracted according to the principles given for the lateral operation.

"The bladder can be washed out by means of a syringe, with a little tepid water. After this, a gum elastic catheter is to be introduced into the bladder through the urethra, and kept in till the adhesive process of inflammation commences, so as to unite the divided surfaces. The patient is to be given an anodyne, composed of about thirty or forty drops of the tincture of opium. He is then to be put to bed, and to be kept lying on his abdomen, till the time above mentioned for removing the catheter. The object I have in view for this last direction must be obvious, namely, to prevent the urine escaping through the incision* into the intestine, which would necessarily retard the union of the parts. By observing the above plan, another grand object is accomplished, that of preventing the urine insinuating itself into the cellular substance. In the natural position of the body, that is, lying on the back, the wounded part of the bladder would be the most depending part; consequently, when the body is prone, the part alluded to is placed in the very reverse position; the urine, dribbling from the ureters, will accumulate in the fundus of the bladder, and pass off by the natural channel through the catheter." (p. 45—49.)

The after treatment, should peritonitis occur, does not, of course, differ from that which would be proposed in the common operation; and Mr. Sleigh considers that his patient ought to be well in six days after the operation.

The next chapter is devoted to an examination of all the objections that our author conceives may be made to the method he proposes, and which he states to be six in number:—1st. The difficulty of reaching the seat of the operation through the anus; 2d, the danger of wounding the peritoneum; 3d, the possibility of an extravasation of urine; 4th, the consequent danger of the wound not healing kindly; 5th, the risk of hemorrhage; and 6th, the difficulty of extracting a calculus of large size through the anus. We have not space to enter into the discussion of these questions: our author obviates them all—to his own satisfaction. The anatomist and surgeon will be able to form an estimate of their weight and value; and some of them are certainly answered by the safe performance of the operation recommended by Vacca, although *this operation* includes the incision of the sphincter ani.

The fourth chapter contains an inquiry into the advantages and disadvantages of the posterior and the lateral methods of performing lithotomy; then follows a view of the anatomy of the pelvic viscera; and afterwards the symptoms of a stone in the bladder are detailed, thus justifying what we hinted above respecting the defective arrangement of the work: since, we conceive, the symptoms of the disease, the compo-

* "It may be observed, 'although the urine be prevented getting into the intestinum rectum by this position, yet it exposes the patient to an evil of equal moment, and what must retard the healing of the incision just as much as the passing of the urine,—namely, the fæces escaping from the intestine into the bladder.' To answer this plausible objection, I must refer my reader to the arguments adopted by me in answer to the third and fourth objections: and also I desire him to remark, I take it for granted that the state of the bowels is attended to, according to the directions given in page 42; by which means the bowels will be so tranquil, that a thousand to one if any fæces will reach the intestinum rectum, before the parts will be, if not healed, at the very least so swollen as to obliterate the incision, and to effectually guard against the possibility of this accident occurring."

sition and varieties of calculi, and a description of the parts included in the operation, would have been the most appropriate mode of opening the subject.

A short chapter (the seventh), consisting of a summing-up of the whole question, concludes the volume.

It will be observed that our author's proposed operation has not yet been carried into effect; and that there is no evidence, as far as we know, of its having been performed on the living subject. Mr. Sleigh has, indeed, evinced the possibility of dilating the anus to the extent of two or three inches, without pain or inconvenience; and a plate and description of Mr. Weiss's instrument for that purpose is annexed to the work. It appears to us to bear a very close resemblance to an instrument, the engraving of which is to be found in AMBROSE PARÉ's works, (folio edition, p. 640,) which he calls a *speculum matricis*. We have only to add, that a plate, representing a vertical section of the male pelvis, and showing the situation of the parts concerned in this operation, and the instruments employed by the author, accompanies the work.

---

